# Analysis of effects of meteorological variables on dengue incidence in Bangladesh using VAR and Granger causality approach

**DOI:** 10.3389/fpubh.2024.1488742

**Published:** 2024-11-28

**Authors:** Md. Jamal Hossain, Nazia Sultana, Anwesha Das, Fariea Nazim Jui, Md. Kamrul Islam, Md. Mijanoor Rahman, Mohammad Mafizur Rahman

**Affiliations:** ^1^Department of Applied Mathematics, Noakhali Science and Technology University, Noakhali, Bangladesh; ^2^Department of Mathematics, Mawlana Bhashani Science and Technology University, Tangail, Bangladesh; ^3^School of Business, University of Southern Queensland, Toowoomba, QLD, Australia

**Keywords:** VAR model, Granger causality, meteorological variables, dengue fever, VECM model, impulse response function

## Abstract

**Background:**

Dengue fever is a serious public health issue in Bangladesh, where its incidence rises with the monsoon. Meteorological variables are believed to be responsible factors among others. Therefore, this study examines the effects of meteorological variables (temperature, rainfall, and humidity) on dengue incidence in Bangladesh. While previous studies have examined the relationship between dengue and meteorological variables using single model approaches, this study employs advanced econometric techniques to capture dynamic interactions. Furthermore, in the case of Bangladesh, this type of analysis is necessary due to the fact that dengue outbreak become one of the major issues. However, the analysis related to this issue is not available.

**Methods:**

For estimation purposes, the Augmented Dickey-Fuller (ADF) test, Vector Autoregressive (VAR) model, Granger causality tests, Impulse Response Function (IRF), Variance Decomposition (VDC), and Vector Error Correction Model (VECM) are employed.

**Results:**

Rainfall has a significant impact on dengue incidence compared to temperature and humidity. The Granger causality test demonstrates that rainfall and dengue incidence are causally related unidirectionally. Rainfall can potentially have a short-term and long-term effect on the incidence of dengue, as per the estimates of the VECM model.

**Conclusions:**

These findings will assist policymakers in Bangladesh in developing a dengue fever early warning system depending on climate change. In order to efficiently avoid the spread of dengue in Bangladesh's dengue-endemic urban areas, this study suggests societal monitoring.

## 1 Introduction

In recent decades, dengue incidence has dramatically surged globally, putting approximately half of the world's population at risk. There is a major health concern to Bangladesh (1) and other countries due to the spread of a mosquito-borne arbovirus that causes dengue fever (DF) from urban to rural areas. Mostly Aedes aegypti and a smaller amount of Aedes albopictus, female mosquitoes carrying the dengue virus are the vectors that spread the infection to people (WHO, 2024^1^). This is a disease that might affect 50% of people on the planet, which affects over 390 million people annually (WHO, 2024, see text footnote^1^). Aedes albopictus also contributes significantly to dengue spread in Europe, and the United States, whereas Aedes aegypti is more prevalent in Asia. High temperatures aid mosquito development, and increased rainfall creates more habitats for vectors (2). Seasonal outbreaks of dengue are common, with higher case counts during the rainier and warmer months. Rainfall and the density of population are other important considerations, as Aedes mosquitoes fancy to lay their eggs in artificial vessels, which are more commonly seen in metropolitan areas (3). Globally, there were 30.67 million DF cases in 1990 and 56.88 million cases in 2019 (4, 5). Comparatively speaking to other nations, over half of the global cases of DF illness are found in South Asian countries, with the Western Pacific, America, and Southeast Asia areas most at risk from DF illness (2). The lengthened rainy seasons and rising temperatures in Southeast Asia's subtropical zones may provide favorable conditions for the growth of Aedes mosquito populations, which transmit dengue (6). Furthermore, there has recently been an increase in dengue incidence reported in temperate regions, such as Nepal (7), indicating that the disease may be moving from subtropical to colder climates and endangering northern parts of India, Pakistan, and the neighboring countries. The first known dengue epidemic in Bangladesh's capital city of Dhaka was described in 1964 (8), and there were occasional instances of dengue fever throughout the years 1977–1978 and 1996–1997, however the magnitude of dengue incidence in Bangladesh was not well documented. In 2019, the country had its biggest-ever epidemic, while the second-biggest one happened in 2018, only a year before. The unprecedented rise in epidemic size in 2018 and 2019 may have been influenced by climate variability, although this is still completely unknown (9). Consequently, there is notable seasonality and year-to-year fluctuation in the number of dengue outbreaks in Bangladesh.

Polwiang (10) examined the epidemiology of dengue and its association with weather circumstances in Bangkok. The findings reveal that Bangkok's dengue transmissibility is positively correlated with humidity and rainfall. A study conducted in Brazil found that using the temperature of the city level as a parameter makes the seasonal pattern of the Aedes mosquito's profusion visible (11). Chaves et al. (12) examined that temperature change significantly alters Aedes mosquito abundance in Thailand. Cheng et al. (13) studied the connection between climate and vector abundance in relation to the threat of dengue outbreaks in Guangzhou, China. They found that while heavy monsoon precipitation enhances the number of vectors, early and frequent intervention together with reduced vertical spread can eventually reduce the probability of a dengue epidemic. According to Tosepu et al. (14), in the Kolaka region the climate has a significant impact on the occurrence of Dengue hemorrhagic fever. It was discovered from a study of historical data that the weather variables, particularly temperature, rainfall, and humidity, have suddenly changed over the last several years (15). Several studies have been performed to investigate the dengue occurrence in Bangladesh using historical climate data (16, 17). It was found in these previous studies that temperature and rainfall were significant contributing factors (18, 19). Zahirul Islam et al. (20) used time series monthly data from 2000 to 2009 to investigate the connection between dengue disease and climatic variability in two major Bangladeshi cities, Dhaka and Chittagong. According to the research, dengue incidence in Dhaka and Chittagong was substantially correlated with the yearly average rainfall and humidity. The association was examined using the ARIMA model. Islam et al. (21) explored how climate influences vector abundance, which has an impact on dengue incidence in Dhaka, Bangladesh. The findings reveal that the impact of average humidity and lag average rainfall on dengue transmission was shown to be considerable. The generalized linear model (GLM) was employed to estimate and analyze the data. Karim et al. (22) discovered that rainfall has the greatest impact on dengue occurrence in Dhaka out of all climatic factors. They used Analysis of Variance (ANOVA) to study the correlation between climate variables and dengue transmission. Aedes profusion was researched by Paul et al. (23) in the city of Dhaka, Bangladesh, focusing solely on the correlation between weather circumstances and vector abundance. They came to the conclusion that rainfall, temperature, and humidity had a substantial impact on the average mosquito abundance.

There has been a significant number of studies done to explore the effect of meteorological variables on dengue incidence using single model approaches; however, none of them consider advanced econometric techniques such as the VAR model, Granger causality tests, Impulse Response function, Variance Decomposition, and VECM for cross-validation and incorporation of significant findings. These models have a very strong ability to identify associations between the variables. Specially previous studies on the influence of dengue occurrence by the meteorological factors in Bangladesh have not incorporated these methodologies.

Bangladesh is a home of dengue incidence. Last year many people died due to this viral fever. Therefore it's important to identify meteorological effects on dengue incidence so that we can make appropriate decisions to minimize risk. The VAR model enables a more thorough understanding of the dynamic interactions between the relevant variables. The Granger causality test captures the causal connections between the variables. Additionally, the use of the VAR model in conjunction with the VECM model approach is also unique to this study. This combination enables us to capture both the short-term interdependencies and the long-term effects of climate factors on dengue incidence. Here, we investigated the impact of meteorological variables on dengue incidence in Bangladesh using a time-series analysis approach. Further, we explore the possibility of the Granger causality methodology to establish causal links between climatic variables and the epidemiology of infectious diseases for the first time to our knowledge. The Granger causality test is used to elucidate underlying causal mechanisms in this model, which is enormously popular in economics.

## 2 Methods

### 2.1 Data collection and study area

The present research includes meteorological and dengue occurrence data spanning 9 years, from 2012 to 2020. Monthly dengue statistics were obtained from Bangladesh's Directorate General of Health Services (DGHS). The Bangladesh Meteorological Department (BMD) provided monthly average temperatures, rainfall, and humidity data. The BMD maintained and managed accumulated weather data at 35 different weather stations across the nation. While humidity is recorded in (%), temperature and rainfall are measured in degrees Celsius (°C) and millimeters (mm), respectively. To obtain monthly data for each station, monthly information for each month was averaged. The monthly averages of temperature, precipitation, and humidity across the country were calculated by averaging the data from all 35 weather stations for the years 2012 to 2020. The data was collected monthly basis due to the fact that the availability of data from BMD and DGHS. Another factor was that dengue incidence were discovered practically year-round in Bangladesh. One probable explanation for this event is because Bangladesh's rainy season lasts roughly 4 to 6 months. Furthermore, on a 1-month scale, monthly averaged climate variable can provide information about monthly dengue incidence.

**Figure 1:**
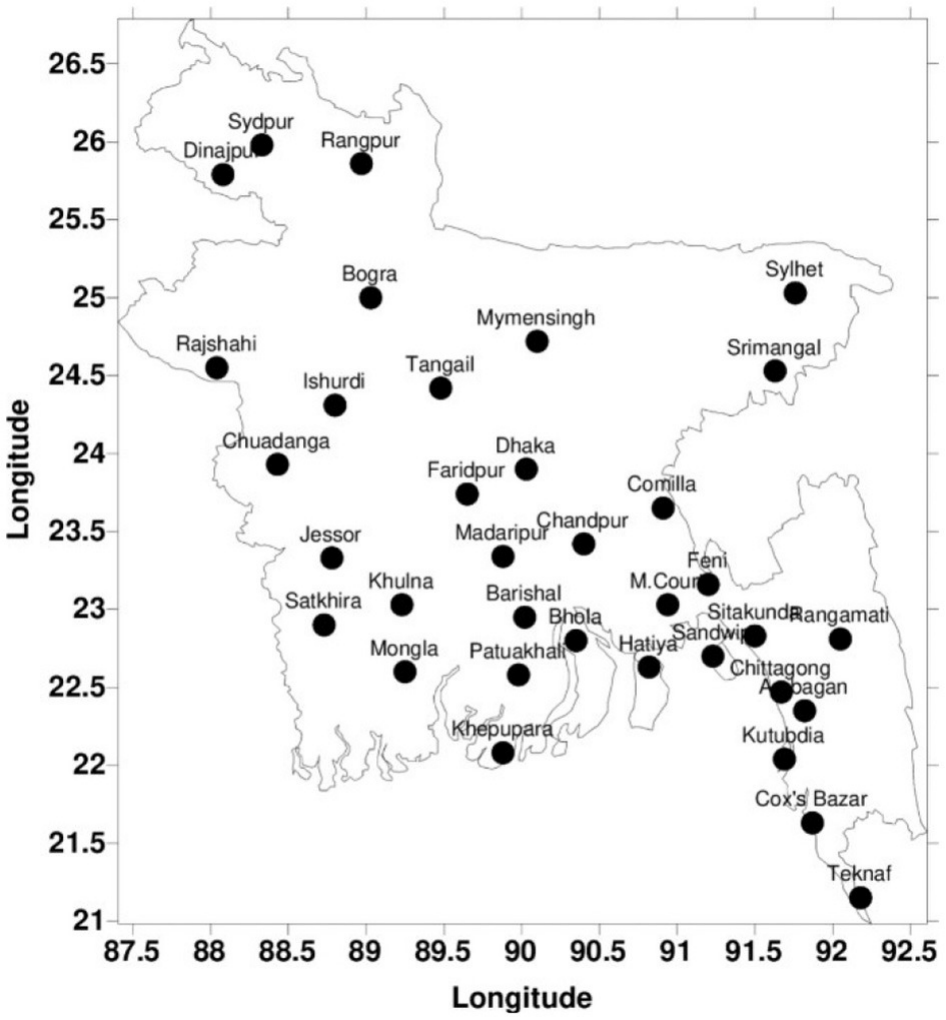
The map located 35 weather stations in Bangladesh (source: BMD).

Bangladesh is located at 20°590 N to 26°630 N and 88°030 E to 92°670 E. When the Tropic of Cancer crosses Bangladesh from east to west, it is at coordinates 23°260 N and 88°470 E. Figure 1 shows the 35 weather stations in Bangladesh.

### 2.2 Stationary time series

According to Nelson and Ploser (39), the majority of time series data have a unit root problem, which skews the results of regression analysis. Additionally, in order to examine the cointegration among the model's variables, the time series data must be stationary. Dengue is the dependent variable in this study, and temperature, rainfall, and humidity are the independent variables. For removing the non-stationarity issue in time series data, this study took first difference and used the ADF (1981) test to eliminate unit root issues. ADF uses an additional lag of dependent variables as an explanatory variable to lessen autocorrelation.

### 2.3 VAR model specification

The Vector Autoregressive (VAR) model is practical, dependable, and simple to modify in multivariate analysis. A method for describing how variables interact over time in a complex multivariate system is the VAR model. It is a dynamic multivariate time series annex to the univariate model. The functional relationship between Dengue, Temp, RF, and HD of Bangladesh can be expressed in the following way:


$$\begin{array}{l} Dengue=f\left(Temp,RF,HD\right) \end{array}$$

The model will test the effect of Temp, RF, and HD on Dengue


$$\begin{array}{l} Dengue_{t}={\beta_{0}+\beta_{1}Temp_{t}+\beta_{2}RF_{t}+\beta_{3}HD}_{t}+\varepsilon_{t} \end{array}$$

where, Dengue = dengue incidence in Bangladesh, Temp = Temperature, RF = Rainfall, and HD = Humidity.

In this model β_0_ is the current time period of the observation of each variable based on the lag values. β_1_, β_2_ and β_3_ are the coefficients of all those independent variables. This paper has been conducted on the following hypothesis:

H0 = Temp has no significant impact on Dengue.
H1 = Temp has a significant impact on Dengue.
H0 = RF has no significant impact on Dengue.
H2 = RF has a significant impact on Dengue.
H0 = HD has no significant impact on Dengue.
H3 = HD has a significant impact on Dengue.

The coefficient of regression, β represents how much the dependent variable changes when the independent variable changes by one unit. The Cholesky decomposition of the contemporaneous covariance matrix served as the foundation for the forecast variance decompositions and impulse responses.

### 2.4 Structural analysis by Granger causality

The Granger causality test was developed by (24) after being introduced by (25). In this study, we will investigate the causal association between dengue, temperature, rainfall, and humidity in the context of Bangladesh. The following technique should be used while using the linear Granger causality tests to determine the causal link between the system's variables. Examine and contrast the unconstrained models:


(1) $$\begin{array}{l} \Delta Y_{t}=\alpha_{1}+\overset{m}{\underset{i=1}{\sum }}\beta_{1i}\Delta Y_{t-i}+\sum_{j=1}^{m}\theta_{1j}\Delta Y_{j-i}+\;e_{1t} \end{array}$$

(2) $$\begin{array}{l} \Delta X_{t}=\alpha_{2}+\overset{m}{\underset{i=1}{\sum }}\beta_{2i}\Delta Y_{t-i}+\sum_{j=1}^{m}\theta_{2j}\Delta X_{j-i}+\;e_{2t} \end{array}$$

with the restricted models:


$$\begin{array}{l} \Delta Y_{t}=\alpha_{1}+\overset{m}{\underset{i=1}{\sum }}\beta_{1i}\Delta Y_{t-i}\\ \Delta X_{t}=\alpha_{2}+\overset{m}{\underset{i=1}{\sum }}\beta_{2i}\Delta Y_{t-i} \end{array}$$

where, Δ*Y*_*t*_ and Δ*X*_*t*_ first-order forward differences of the variables; α, β, and θ are the parameters to be estimated; and *e*_1_ and *e*_2_ are standard random errors. If statistically θ_1_ is significant and θ_2_ is not, then changes of *Y*_*t*_ Granger causes changes of *X*_*t*_ or vice-versa. Bivariate causality exists between the variables if both of them are statistically significant; if insignificant, neither changes of *Y*_*t*_ nor changes of *X*_*t*_ have any consequence on other variables.

### 2.5 Statistical analysis

The Vector Autoregressive model (VAR) method was applied in this research to investigate the relationship between meteorological factors and the occurrence of dengue. One of the most adaptable models for multivariate time series analysis is VAR. The main benefit of VAR is the simultaneous explanation and explanatory nature of multivariate variables. As a result, this model makes predictions that are more accurate by taking into account the relationships between various variables. This model, which uses the Granger causality test to explain underlying causal mechanisms, is very well-liked in the field of economics. Additionally, this study used the IRF to pinpoint shock responses to changes in temperature, rainfall, and humidity. IRF monitors how each variable affects the other variables in the system. Variance decomposition (VDC) was employed in order to forecast the incidence of dengue incidence in the upcoming year. Lastly, we use the vector error correction model (VECM) to discover a long-term correlation between weather variables and dengue incidence.

## 3 Results

**Figure 2:**
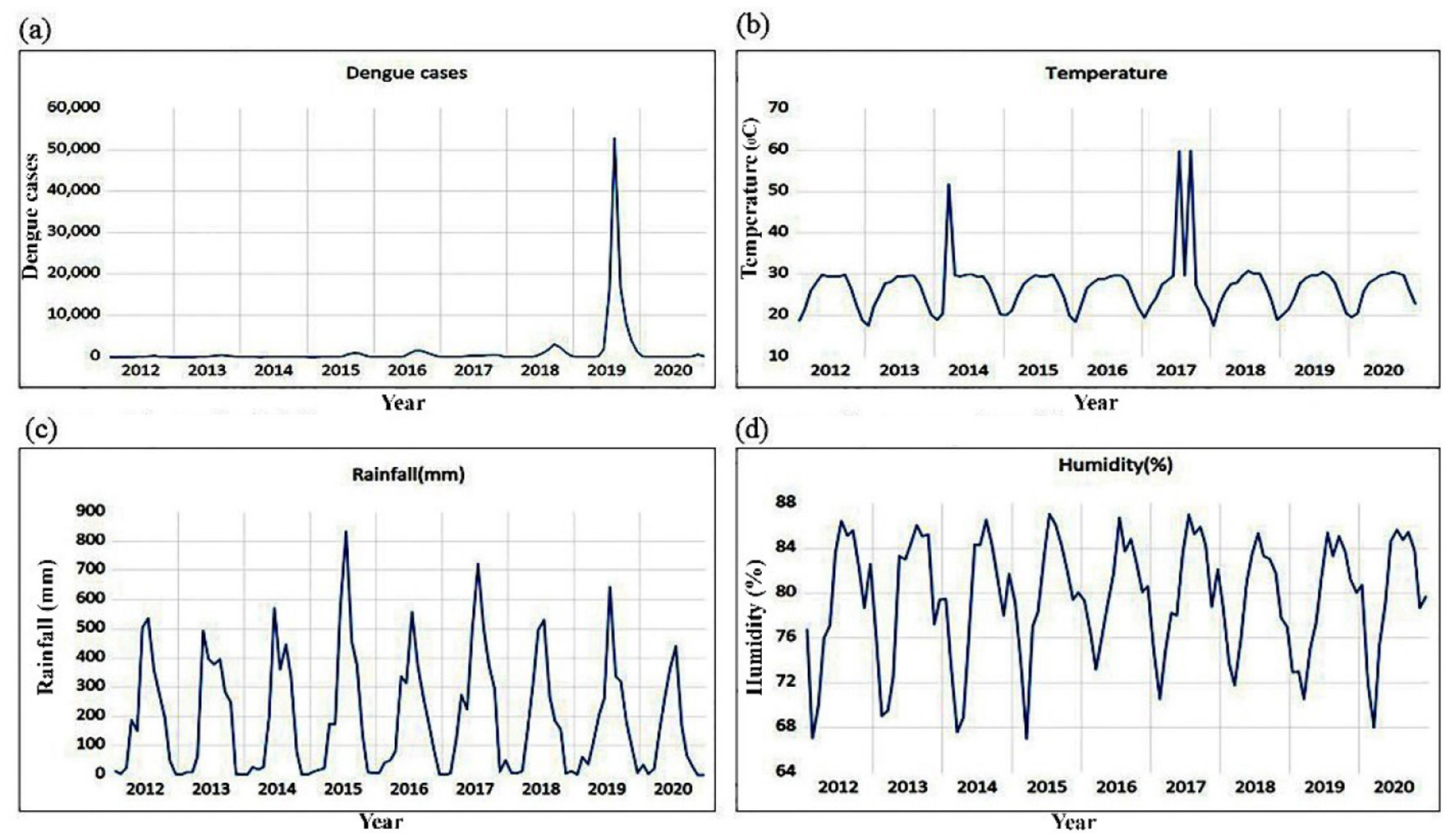
The Time series plot of monthly dengue incidence, temperature, rainfall, and humidity from January 2012 to December 2020. **(a)** Number of dengue cases. **(b)** Monthly average temperature. **(c)** Monthly total rainfall. **(d)** Monthly average humidity.

Dengue fever transmission in Bangladesh typically starts in June, peaks in July and August then starts to decline gradually in October (90 cases), reaching its lowest point by December in 2012. The time series plot of dengue incidence, temperature, rainfall, and humidity has been shown from January 2012 to December 2020 in Figure 2. A total of 127,693 dengue incidence were reported in Bangladesh from 2012 to 2020. The maximum (101,354) number of cases was reported in 2019, while the minimum (375) number of cases was obtained in 2014. Contrary to the total monthly cases, which indicates that September had the highest number of dengue incidence reported, the highest monthly number of dengue incidence (52,636 cases) was reported in August 2019. The monsoon season (June to September) has the highest number of dengue incidence each year (38,087 cases), followed by the summer/pre-monsoon season (February–May) and the winter/post-monsoon season (October–January) with 11,032 cases each.

The descriptive statistics of monthly weather variables and dengue incidence are shown in Table 1. Dengue incidence have a mean of 1,182.343, standard deviation (SD) of 5,543.198, minimum of 3,162, and maximum of 52,636. The largest number of cases in Bangladesh is 52,636. The monthly averages for temperature, rainfall, and humidity are 27.02°C, 191.98%, and 79.57 mm respectively, while the average number of confirmed cases is ~1,182.343. According to this study's descriptive statistic, the lowest temperature recorded was 17.39°C, while the maximum recorded temperature was 31.86°C. Furthermore, a low of 175.05 mm and a high of 432.2 mm of rainfall are recorded.

**Table 1:** Descriptive statistics of monthly weather variables and dengue incidence.

**Variables**	**Numbers of months**	**Mean**	**SD**	**Minimum**	**Maximum**
Dengue	108	1,182.343	5,543.198	3,162	52,636
Temperature	108	27.01662	6.421443	17.39	31.86
Rainfall	108	191.9788	197.7561	175.05	432.2
Humidity	108	79.57204	5.256397	41.35	91.52

According to the ADF test results (in Table 2), all variables are non-stationary at the level. All the variables are found stationary after the first difference; thus we accept the alternative hypothesis in place of the null hypothesis.

**Table 2:** Augmented Dickey-Fuller (ADF) test for unit root.

**Variables**	**Test statistic**	**1% critical value**	**5% critical value**	**10% critical value**	***p*-value**	**Remarks**
Dengue	−1.381	−3.508	−2.890	−2.580	0.4410	Non-stationary
dDengue	−11.653	−3.508	−2.890	−2.580	0.0000	Stationary
Temperature	−1.588	−3.508	−2.890	−2.580	0.4897	Non-stationary
dTemperature	−17.874	−3.508	−2.890	−2.580	0.0000	Stationary
Rainfall	−1.917	−3.508	−2.890	−2.580	0.3239	Non-stationary
dRainfall	−9.242	−3.508	−2.890	−2.580	0.0000	Stationary
Humidity	−1.953	−3.508	−2.890	−2.580	0.3075	Non-stationary
dHumidity	−8.737	−3.508	−2.890	−2.580	0.0000	Stationary

OLS regression analysis is not applicable when all of the variables are correlated. Temperature, Rainfall, and Humidity were serially correlated, as shown by the Durbin-Watson statistics (DW = 0.939). Low Durbin Watson statistics show a positive serial correlation between variables and dengue incidence. Both the Breusch-Godfrey test (Prob > chi2 = 0.000) and Durbin's alternative test (Prob > chi2 = 0.000) were used to find serial correlations. The Breusch-Godfrey test (Prob > chi2 = 0.000) and Durbin's alternative test (Prob > chi2 = 0.000) both detected serial correlations in the analysis of OLS regression using the lag model. OLS regression analyses were therefore not appropriate for this study. As a result, the VAR model was used due to the presence of serial correlations.

### 3.1 VAR model

Choosing the true lag lengths has a significant influence on the forecasting accuracy of VAR models. The number of prior observations that are utilized to forecast the variable's present value is referred to as the lag length. Likelihood Ratio (LR), Final Prediction Error (FPE), Akaike Information Criterion (AIC), Schwarz information standard (SC), and the Hannan Quinn (HQ) information standard were used to determine the ideal latency. This analysis shows that the order five lag length yielded the LR, minimum FPE, and AIC value when compared to other order lag lengths. The outcomes of the selection criterion are displayed in Table 3.

**Table 3:** VAR(p) model lag selection criterion.

**Lag**	**LR**	**FPE**	**AIC**	**SC**	**HQ**
0	Na	3.56e+14	44.85660	44.96145^*^	44.89902
1	63.99158	2.49e+14	44.49907	45.02334	44.71119
2	33.32500	2.38e+14	44.45203	45.39571	44.83384
3	48.57521	1.88e+14	44.21043	45.57352	44.76194
4	57.45117	1.30e+14	43.83304	45.61555	44.55424^*^
5	30.46479^*^	1.23e+14^*^	43.76570^*^	45.96762	44.65660
6	20.13399	1.31e+14	43.81685	46.33818	44.87744
7	17.13895	1.46e+14	43.89524	46.93599	45.12553
8	20.42294	1.53e+14	43.90903	47.36919	45.30902

^*^Denotes significance at 10% significance levels.

Since there is a serial correlation of up to five lags, we can reject the null hypothesis and accept the alternative hypothesis. As a result, we made the choice to employ a VAR model with an order-five lag. The degree to which past data might impact the future results of the variables is indicated by the lag order of a VAR model. In this case, the fifth lag indicates that meteorological factors from the preceding 5 months will affect the incidence of dengue in the future. This finding is supported by Figure 2, which shows that dengue incidence in Bangladesh is present nearly year-round. Bangladesh experiences 4–6 months of rainy season. Aedes mosquitoes breed during this time, lay their eggs, develop during this time, and continue to spread dengue incidence.

#### 3.1.1 Estimation of the VAR model

The VAR model is used to predict the relationship that affects each other. *p*-value for the 5% level of significance is 0.05. If this value is <0.05 the variable at lag 1, 2, 3, 4, and 5 is significant. If this value is >0.05 the variable at lag 1, 2, 3, 4, and 5 is insignificant at that lag length.

Based on the findings of the *p*-value (in Table 4), the VAR model results demonstrate that dengue is not greatly influenced by temperature or humidity. Additionally, the results show that rainfall has a notable influence on dengue incidence in Bangladesh. This means that rainfall has more influence on Dengue counter to temperature or humidity. From a practical standpoint, this indicates that Aedes mosquitoes develop, lay their eggs, hatch, mature, and propagate incidents of dengue during the rainfall increase. However, there are additional elements that contribute to the occurrence of dengue, including still water, inadequate sanitation, mosquitos' bites, and population density. Since meteorological factors greatly affect the Aedes mosquitos' population which then affect the incidence of dengue, we wanted to examine meteorological factors effects here. We discovered that rainfall significantly affects the incidence of dengue.

**Table 4:** Estimated results from the VAR model.

**Variables**	**Coefficient**	**Standard error**	***z*-ratio**	***p*-value**
**Temperature**				
Lag 1	−16.6288	91.307	−0.18	0.855
Lag 2	70.876	113.39	0.63	0.532
Lag 3	101.833	107.55	0.95	0.344
Lag 4	27.280	108.07	0.25	0.801
Lag 5	53.511	92.73	0.58	0.564
**Rainfall**				
Lag 1	18.4631	5.3844	3.43	0.001
Lag 2	−4.696	5.1403	−0.91	0.361
Lag 3	1.631	5.5437	0.29	0.769
Lag 4	−6.837	5.5469	−1.23	0.218
Lag 5	−2.473	5.5419	−0.45	0.655
**Humidity**				
Lag 1	−180.4344	196.665	−0.92	0.359
Lag 2	208.8375	197.28	1.06	0.290
Lag 3	77.3843	198.370	0.39	0.696
Lag 4	323.5588	178.646	1.81	0.070
Lag 5	194.5566	203.747	0.95	0.340
Constant	−67.87002	450.615	−0.15	0.880

**Figure 3:**
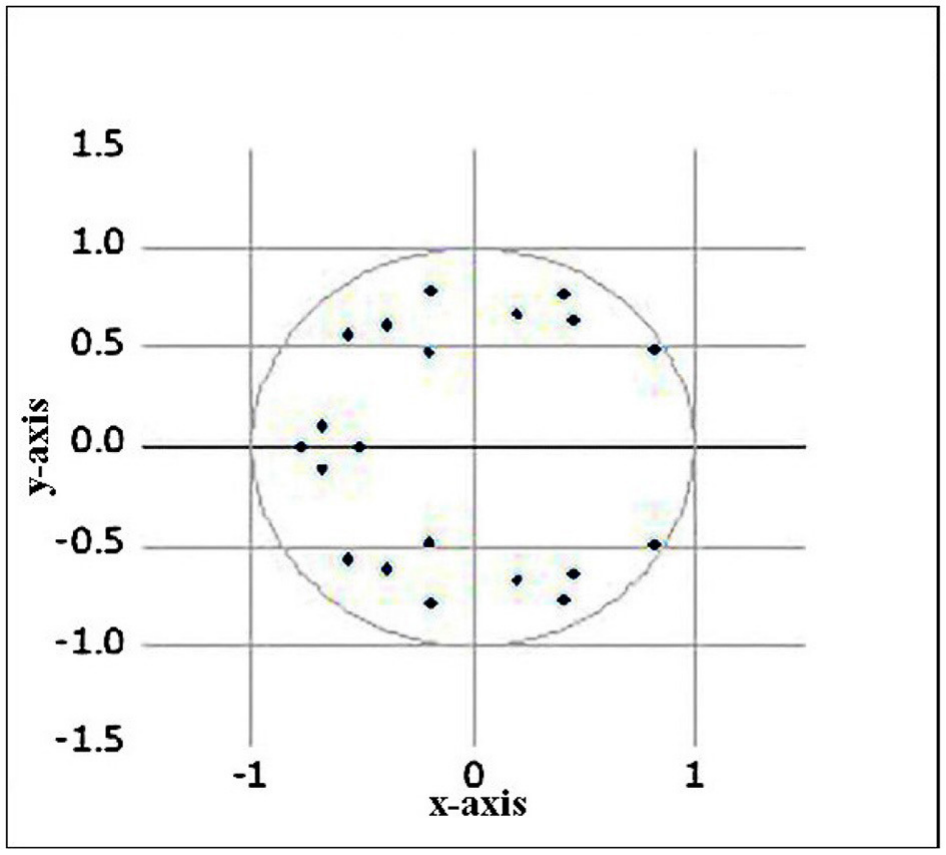
Inverse root of AR characteristic polynomial.

The VAR system must be stationary to become stable. Figure 3 shows the Inverse Root of Auto Regression (AR) Characteristic Polynomial using a complex coordinate system. AR roots are used to report the inverse root of the AR polynomial's properties. The estimated VAR is stable if every root of the characteristic AR polynomial has a modulus of less than one and lies inside the unit circle. If any of the estimated roots have a modulus greater than one and are outside the unit circle the estimated VAR is not stable. Since no root lies outside the unit circle, therefore, VAR satisfies the stability condition. This means that the assumption of this study is right and produced accurate results.

### 3.2 The result of Granger causality test

The Granger causality technique was used to study the formation of the causal associations among the variables (26, 27). If the probability value falls below a certain α level, the hypothesis is rejected. The Granger causality test allows for only two variables to be investigated at once.

Ho: *X*_*t*_ does not Granger cause *Y*_*t*_.

If the *p*-value is >0.05, accept Ho. It means no causality.

If the *p*-value is <0.05, reject Ho. It means causality exists.

The Granger causality test was used in this study to identify the causal connection between climate conditions and dengue incidence, as shown in Table 5. The Granger causality test confirms that rainfall in Bangladesh affects the prevalence of dengue. Consequently, rainfall plays a significant role in dengue prevalence in Bangladesh. The VAR-Granger supports the idea that rainfall causes dengue to occur in Bangladesh.

**Table 5:** Pair-wise Granger causality test results.

**Null hypothesis**	***F*-Statistic**	**Probability**	**Decision**
HD does not Granger cause DENGUE	0.11231	0.8939	No causality
DENGUE does not Granger cause HD	0.16540	0.8478	
RF does not Granger cause DENGUE	6.79116^*^	0.0017	RF → DENGUE (Unidirectional causality)
DENGUE does not Granger cause RF	0.06758	0.9347	
TEMP does not Granger cause DENGUE	1.35082	0.2637	No causality
DENGUE does not Granger cause TEMP	0.42810	0.6529	

^*^Denotes significance at 10% significance levels.

**Figure 4:**
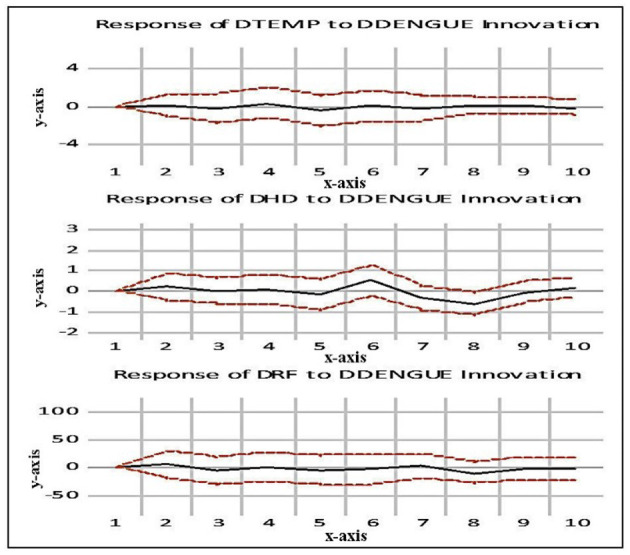
Impulse response function.

A shock's impact on a series' behavior is tracked over time using an impulse response function (28). The period is indicated on the *x*-axis, and the percentage variation is indicated on the *y*-axis. The black line represents the impulse response function, and the red line represents the confidence interval. IRF monitors the impact of a shock of one standard deviation on the present and future values of the endogenous variable for one of the innovations. The results of the IRF are displayed in Figure 4.

### 3.3 Interpretation of a standard deviation (SD) shock to dengue

- *Response on temperature (Temp):* A one SD shock (innovation) to Temp has no appreciable effects on dengue in periods 1 through 10. Be aware that there have been relatively few advancements in dengue over the years. That means there has been little influence over the course of time.
- *Response on humidity (HD):* A one SD shock (innovation) to HD in periods 1 and 4 has no appreciable impact on dengue. The response stays in the negative region from the seventh period to roughly the eighth period, though with increasing tendencies, before increasing sharply in the positive region from the fourth period until the sixth period, when it reaches its steady-state value. This indicates both the immediate and long-term impacts of dengue shocks would be varied.
- *Response on rainfall (RF):* A one SD shock (innovation) to RF in periods 1 and 7 has no appreciable impact on dengue. Beginning in the eighth period and continuing until the ninth period, the response gradually declines in the negative region until it reaches its steady state value, however with stronger tendencies. This suggests that the short and long-term impacts of dengue shocks would vary.

The variance decomposition (VDC) of the forecast error yields the proportion of each variable's unexpected variation that is caused by shocks from other variables. It demonstrates the relative impact of one variable on another. The results of the VDC are shown in Table 6.

**Table 6:** Estimated results using the VDC method.

**Variance decomposition of dengue**				
**Period**	**Standard error**	**Temperature**	**Humidity**	**Rainfall**
1	5,081.234	0.000000	0.000000	0.000000
2	5,471.425	0.333033	1.691872	8.458025
3	5,817.624	0.407248	1.551124	12.31113
4	5,839.749	0.625178	1.685690	12.26568
5	5,843.276	0.693874	1.686830	12.26130
6	5,878.707	0.820317	2.556840	12.28947
7	5,892.172	0.823477	2.550356	12.25050
8	5,913.360	0.820392	2.797026	12.20005
9	5,933.140	0.889799	2.787868	12.51933
10	5,939.771	0.951484	2.830274	12.55278

To find the amount of the forecast error variance for each explanatory variable in the model that may be accounted for by innovations, the VDC is computed over a 10-year prediction horizon. The VDC results demonstrate how well the explanatory variables explain the data. In the short run, over a period of 2 years, temperature, humidity, and rainfall can each independently explain 0.33%, 1.69%, and 8.45% of the variation in dengue influence. Therefore, rainfall is better able to account for variations in the dengue influence over the short term. Again, over a 10-year (long-term) period, temperature, humidity, and rainfall are all responsible for varying amounts of dengue effect, with each factor accounting for 0.95%, 2.83%, and 12.55% of the fluctuation. We may conclude that rainfall in Bangladesh has more ability to account for both the immediate and long-term consequences of dengue.

The VECM also evaluates the robustness of the VAR model's baseline results; the results are shown in Table 7.

**Table 7:** The estimated results of the VECM model.

**Variables**	**Coefficient**	**Standard error**	***z*-ratio**	***p*-value**
Temperature	−21.95	119.40	−0.18	0.854
Rainfall	28.02^***^	10.89	2.57	0.010
Humidity	−397.38	258.98	−1.53	0.125
Error correction term (−1)	−0.42396^***^	0.15034	−2.82	0.005

^***^Denotes significance at 1% significance levels.

The results reveal that the Error Correction Term (ECT) is negative (−0.42) and significant as the short-term shock is corrected over time. The fact that the value is in the range of 0 to −1 suggests that the equilibrium criterion is satisfied by the error correction process. The outcomes of the VECM model demonstrate that there are short-term connections between dengue-temperature and dengue-humidity. Rainfall can also have a long-term impact on the incidence of dengue. Therefore, even though the level of significance varies, we can say that the results of the VECM are trustworthy and largely correspond to those of the primary VAR model.

## 4 Discussion

The health burden of dengue fever has been rising globally, including in South and Southeast Asian nations. The incidence of dengue is caused by female Aedes aegypti mosquitoes carrying the dengue virus, which they then transmit to humans. Since mosquito bites are the primary cause of dengue, both biological and physical phenomena play a significant role in its spread. To incorporate these two phenomena in a single model is complicated. Thus, we simply take into account the influence of physical phenomena (such as meteorological variables). Previous research has been done in these areas to look into how climate change may affect the spread of dengue fever (10, 29). Rainfall, temperature, and humidity were the most frequently utilized weather predictors in these studies that may have an impact on dengue outbreaks. These studies demonstrated the cumulative impact of various climate variables over time. In contrast, this study uses time series analysis to show how meteorological factors affect dengue incidence in Bangladesh. The conclusion drawn from this study's analyses is that dengue incidence in Bangladesh was significantly influenced by total rainfall. The results of this study are almost identical to earlier studies' conclusions, which point to a connection between dengue incidence and meteorological conditions. The majority of previous studies argue that temperature or rainfall, especially increased rainfall, influences the prevalence of dengue. However, the study area and country have an impact on these results. Similarly, the current study shows that there is a significant effect on dengue incidence and rainfall. According to numerous studies, the impact of rainfall on the incidence of dengue can vary throughout the year (30). These findings illustrate that rainfall has the greatest impact on dengue incidence in Bangladesh out of all climatic parameters. Karim et al. (22) revealed similar findings in Dhaka, Bangladesh. According to the concept, the effect of rainfall on the risk of contracting DF changes according to the amount of rainfall (31). When there is no rain, the environment of the drought offers fewer places for eggs to breed, and the mosquito population decreases. However, as new dwelling locations become available after a moderate quantity of rainfall, the vector population will grow. Cheng et al. (32, 33) found that DF was generally positively correlated with intense rainfall; the research carried out in Barbados (34) produced findings that were consistent with this. Both Johansson et al. (35) and Arcari et al. (36) in Puerto Rico and Indonesia noted a significant positive correlation between rainfall and dengue occurrence. In the past, studies from Sri Lanka and Bangladesh (37) indicate both short and extensive lag times among weather conditions and enhancing dengue occurrence.

Our study had two significant strengths. Firstly, this study investigated the effect of meteorological variables on dengue occurrence in Bangladesh using a time-series analysis approach. The VAR model can flexibly examine the dynamic interaction between a number of time series variables. VAR is widely used in time series forecasting and in analyzing the dynamic impact of random shocks on connections of variables. Because all the variables are regarded as endogenous, this is a straightforward model without any exogenous or endogenous complexity. Secondly, our findings revealed an unidirectional connection between rainfall and dengue incidence, according to the Granger causality test, which is unreported in earlier investigations.

## 5 Conclusion

The fastest-moving vector-borne illness in the world is dengue (38). In conclusion, this study's main objective is to investigate the effect of meteorological variables on dengue occurrence in Bangladesh. The VAR model indicates that there is a strong relation between rainfall and dengue occurrence in Bangladesh. Otherwise, neither the temperature nor the humidity have a substantial influence on the incidence of dengue. Further, The Granger causality analysis reveals that there is a unidirectional association between rainfall and dengue incidence. Rainfall consequently has a large impact on dengue incidence in Bangladesh. The findings of VDC, IRF, and VECM suggest that rainfall is the most plausible explanation for the short and long-term effects of dengue incidence in Bangladesh. The study's conclusions will be useful for establishing a weather-based dengue early warning system in Bangladesh for policymakers and practitioners. Additionally, this study advises municipal-based supervision for creating a successful dengue prevention tactic in Bangladesh's dengue-endemic cities. Researchers, planners, and public health authorities will find these findings useful for effectively managing services and setting up the necessary medical infrastructure.

This approach has typically been used to identify a causal relationship between variables. Although this study has implications, it also has some drawbacks. For example, the dynamic aspect of the model may not be well-covered because of the lack of weekly availability of data. Data are only available monthly, which has been used in the study. Furthermore, at the time of this study, monthly weather data for 2021 and 2022 were not made publicly available. Consequently, they weren't incorporated into the models. Due to the fact that migration data were not supplied at monthly intervals, we were not able to incorporate information on immigration-to-emigration ratios or density of population in our time-series analysis. The regional and other complicated variables that affect dengue incidence are likely too complicated for meteorological data to fully explain. In order to further analyze the connections between climatic variables and dengue fever at a larger scale and for various Asiatic countries, future work will make extensive use of Granger causality on a much larger Asian dataset. Future research is advised to take socio-demographic factors like population growth, migration or travel rates, and water storage practices into account when examining the relationship between dengue incidence.

## Data Availability

The raw data supporting the conclusions of this article will be made available by the authors, without undue reservation.

## Footnotes

^1^ https://www.who.int/news-room/fact-sheets/detail/dengue-and-severe-dengue
